# Reflectance and photophysical properties of rhodamine 6G/2-(4-methyl-2-oxo-2H-chromen-7-yloxy) acetic acid as cold hybrid colorant

**DOI:** 10.1038/s41598-022-10001-9

**Published:** 2022-04-12

**Authors:** Behnam Gheitarani, Marzieh Golshan, Mahdi Salami Hosseini, Mehdi Salami-Kalajahi

**Affiliations:** 1 Faculty of Polymer Engineering, Sahand University of Technology, P.O. Box 51335-1996, Tabriz, Iran; 2 Institute of Polymeric Materials, Sahand University of Technology, P.O. Box 51335-1996, Tabriz, Iran

**Keywords:** Chemistry, Materials science, Optics and photonics

## Abstract

Rhodamine 6G (Rh6G) is modified by ethylenediamine to obtain rhodamine with amine functional groups (Rh6G-NH_2_). Rh6G-NH_2_ as an initial core is used to bond coumarin derivatives. Synthesized fluorescent colorants are specified using Fourier transform infrared spectroscopy (FT-IR), proton and carbon nuclear magnetic resonance (^1^H NMR and ^13^C NMR), X-ray diffraction (XRD), and field emission scanning electron microscopy (FE-SEM) to analyze the structure of the fluorescent pigments. Fluorescence microscopy, fluorescence spectrophotometer, and UV–visible–NIR reflectance spectra are used to demonstrate the optical properties. UV–Vis–NIR reflectance spectra showed that synthesized colorants were transparent in NIR region. Also, photophysical properties of 2-(4-methyl-2-oxo-2H-chromen-7-yloxy) acetic acid (MOHCYAA), Rh6G-NH_2_, and hybrid 2-(4-methyl-2-oxo-2H-chromen-7-yloxy) acetic acid/rhodamine 6G (HMR) were investigated. Type of solvent had a strong effect on quantum yield. Rh6G-NH_2_ (*ϕ*_*s*_ = 0.66) and HMR (*ϕ*_*s*_ = 0.72) displayed the maximum quantum yield in ethanol due to good interaction with ethanol and the formation of ring-opened amide form of rhodamine group. Finally, Rh6G-NH_2_ and HMR displayed the maximum quantum yield in ethanol due to good interaction of structure with ethanol and the formation of ring-opened amide form of rhodamine group in compound.

## Introduction

UV light accounts for a small part of energy emitted by the sun, and the most of the sun's energy lies in the visible and near-infrared (NIR) region^1,2^. Manipulation in the visible region changes color of materials, and for a fixed color, the amount of energy absorbed by material in the visible region cannot be changed. Therefore, only way to control amount of energy absorbed by the material exposed to sunlight is to control the absorption of the NIR region, which also contains the most energy emitted from the sun (52%)^3–5^. Paints that have little absorption in the NIR region are called cold paints. These colors, when exposed to sunlight, reflect NIR waves, dissipating energy, and keeping their temperature low^6,7^. This has received a great deal of attention from scientists because existence of such properties has very interesting and important applications. Fluorescent pigments, which are a member of the photoluminescent materials category, are capable to convert the absorbed visible or ultraviolet light to the specific color of visible light owing to the high intensity of reflected light property. Some fluorescent paints, such as coumarin and rhodamine, have often advanced conjugation systems and a number of hybrid rings. Coumarin derivatives due to light stability, high quantum fluorescence efficiency, and low toxicity are widely used as dyes for applications in organic electroluminescence diodes^8,9^, sensor chemistry^10^, micelles^11^, antimicrobial^12^, and imaging^13^. Also, coumarin derivatives have expanded emission spectrum that can be adjusted from blue to NIR via changing donors^14^. Ferasat et al.^15^ synthesized the fluorescent coumarin/perylene-3,4,9,10-tetracarboxylic diimide hybrid. The fluorescence properties of the synthesized dyes and their application as cold dyes were discussed. Furthermore, the synthesized dyes were belong to transparent category in NIR region. Rhodamine derivatives are another fluorescent dyes that can be used as laser dye due to their unique structure, photochemical properties, photostability, and high quantum efficiency^16,17^. Considering the spirolactam ring-opening structure of rhodamine, it can be a suitable choice for detecting pH values because spirolactam structure possesses two different states in acidic and basic pH media. It is non-fluorescent in basic media while it shows strong fluorescence emission in acidic media. It is noteworthy that evaluation of pH is substantial in biological, chemical, and industrial fields^18–20^. Among all the systems studied in early years, fluorescent probes were chosen as a good candidate to be used as pH detection system on account of high sensitivity, selectivity, and potential use in many fields^21–23^. However, due to the unique properties of rhodamine-based pH probes, they have got considerable attention as dual-switch pH sensors and cold pigments^24–26^. Amani et al.^27^ investigated the photophysical and reflective properties of perylene-3,4,9,10-tetracarboxyl diimide (PTCDI)/rhodamine 6G hybrid for use in cold colors. Fluorescence quantum yield of PTCDI-Rh6G hybrid was investigated in different solvents and the highest efficiency was obtained 0.27 in DMF solvent. They found that synthesized fluorescent dyes were classified as transparent and adsorbent dyes in the NIR region.

Heretofore, the effect of rhodamine 6G on the structure of dye and hybridization with coumarin has not been studied in scientific sources. This has a significant impact on the cold paint industry. The purpose of this work is synthesis and investigation of optical and NIR reflectance properties of hybrid rhodamine 6G-coumarin dye. Rh6G-NH_2_ is prepared by modification of rhodamine 6G (Rh6G) by ethylenediamine. Then, amine-functionalized rhodamine is used as core and 2-(4-methyl-2-oxo-2H-chromen-7-yloxy) acetic acid is reacted to amines via amidation reaction to prepare rhodamine 6G-2-(4-Methyl-2-oxo-2H-chromen-7-yloxy) acetic acid hybrid (HMR). Finally, NIR reflectance and photophysical behaviors under different conditions are investigated.

## Experimental methods

### Synthesis of amino-functionalized rhodamine 6G (Rh6G-NH_2_)

**Scheme 1:**
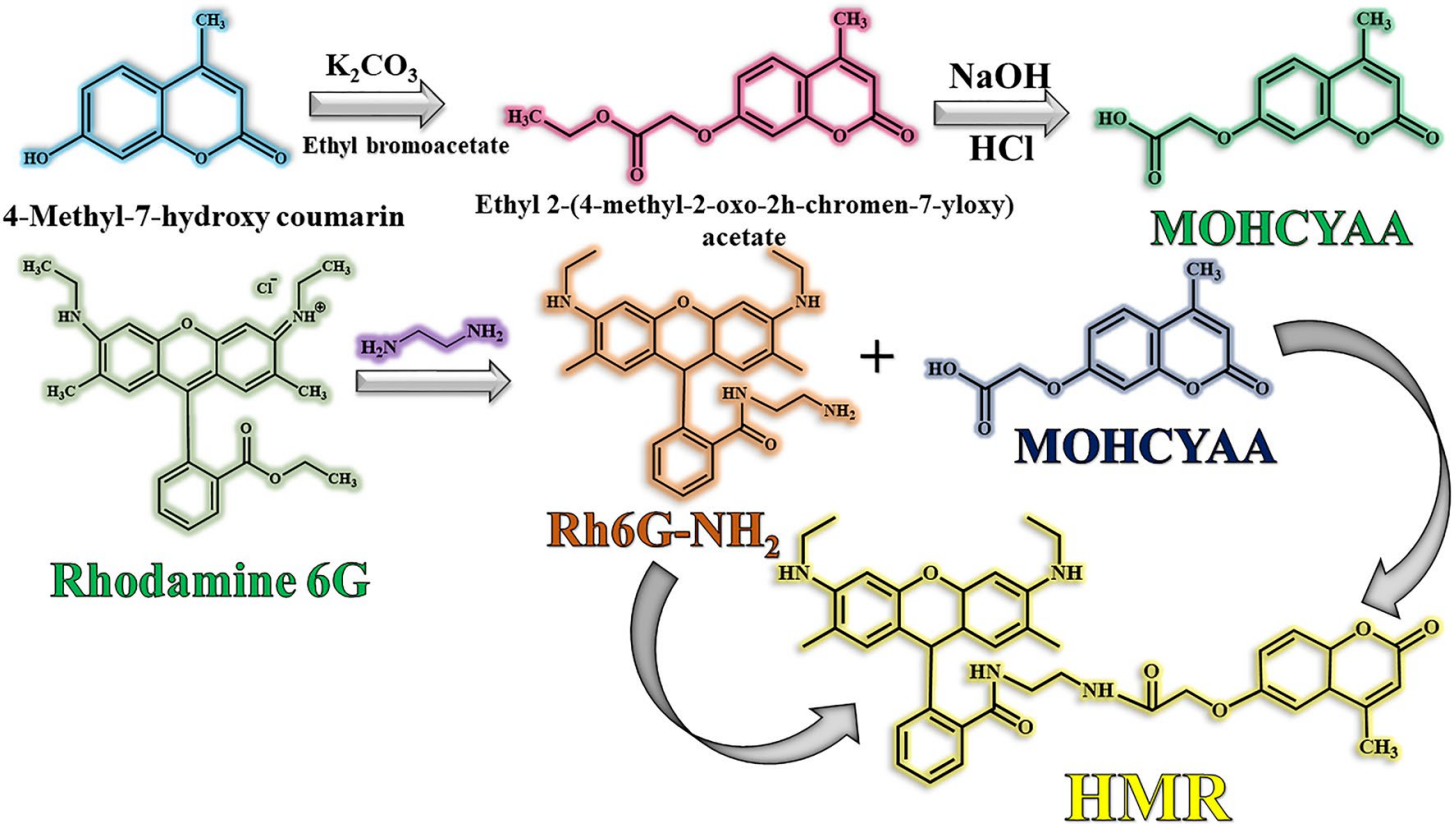
The synthetic route of HMR pigment (modified Rhodamine 6G).

To prepare the luminescent core (Scheme 1), 2.3 g (4.6 mmol) rhodamine 6G (Rh6G) was dissolved in 90 mL ethanol. Then, 1.8 mL (28 mmol) ethylenediamine (EDA) was added and the reaction was performed for 5 h at 65 °C. After reaction completion, mixture was diluted with 25 mL distilled water and filtered. Finally, amino-functionalized rhodamine 6G (Rh6G-NH_2_) was dried in a vacuum oven at 65 °C for 24 h^28^. The yield of reaction was gravimetrically obtained ~ 88%.

**Figure 1:**
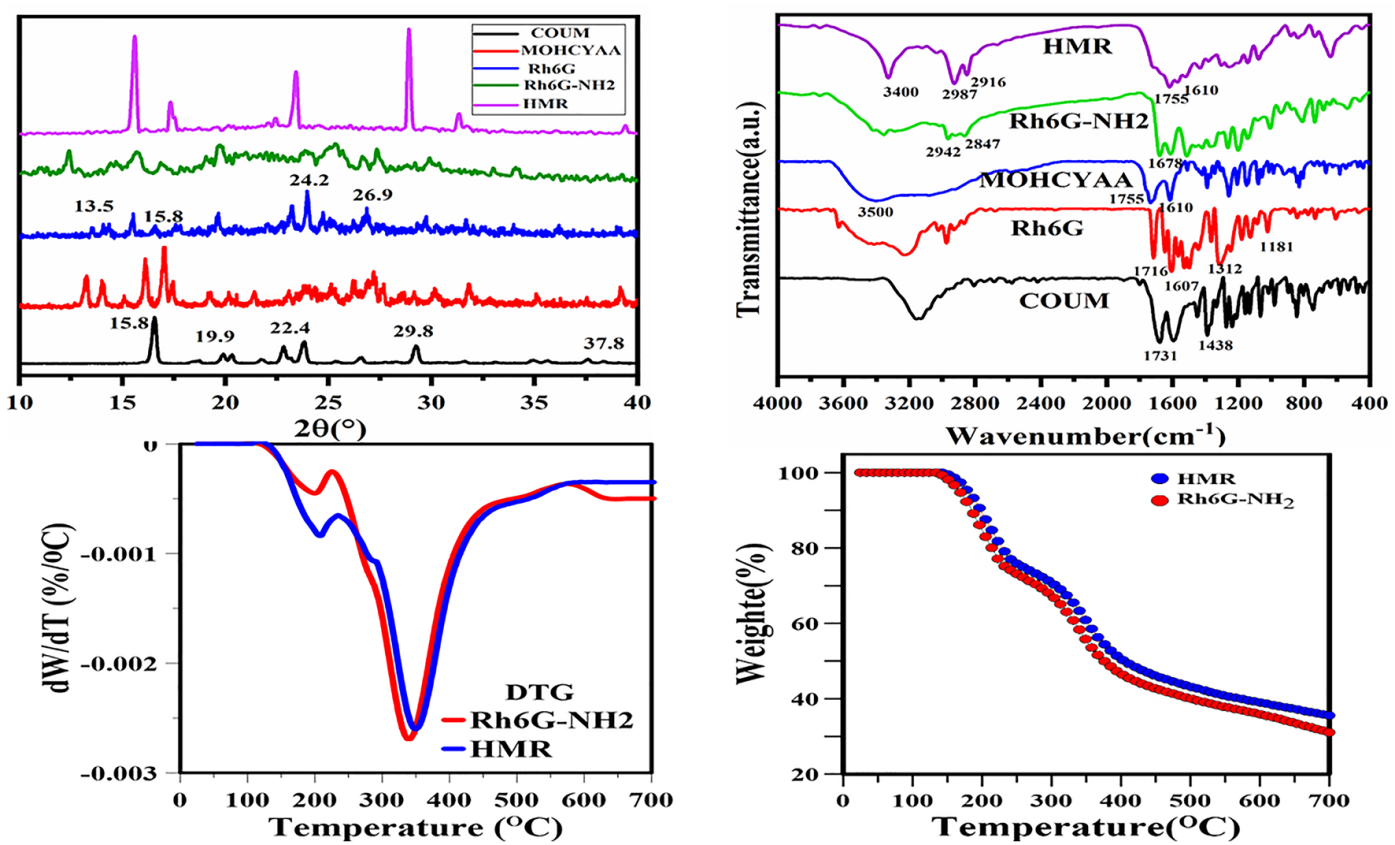
FTIR spectra, XRD patterns, TGA, and DTG thermograms of different samples.

FT-IR main absorption peaks of Rh6G-NH_2_ (KBr, υ/cm^−1^, Fig. 1): 3192 (υNH_2_), 3410 (υNH), 2942, 2848 (νCH); 1678 (νC = O); 1634, 1528 and 1484 (νAr = CH)^28^.

**Figure 2:**
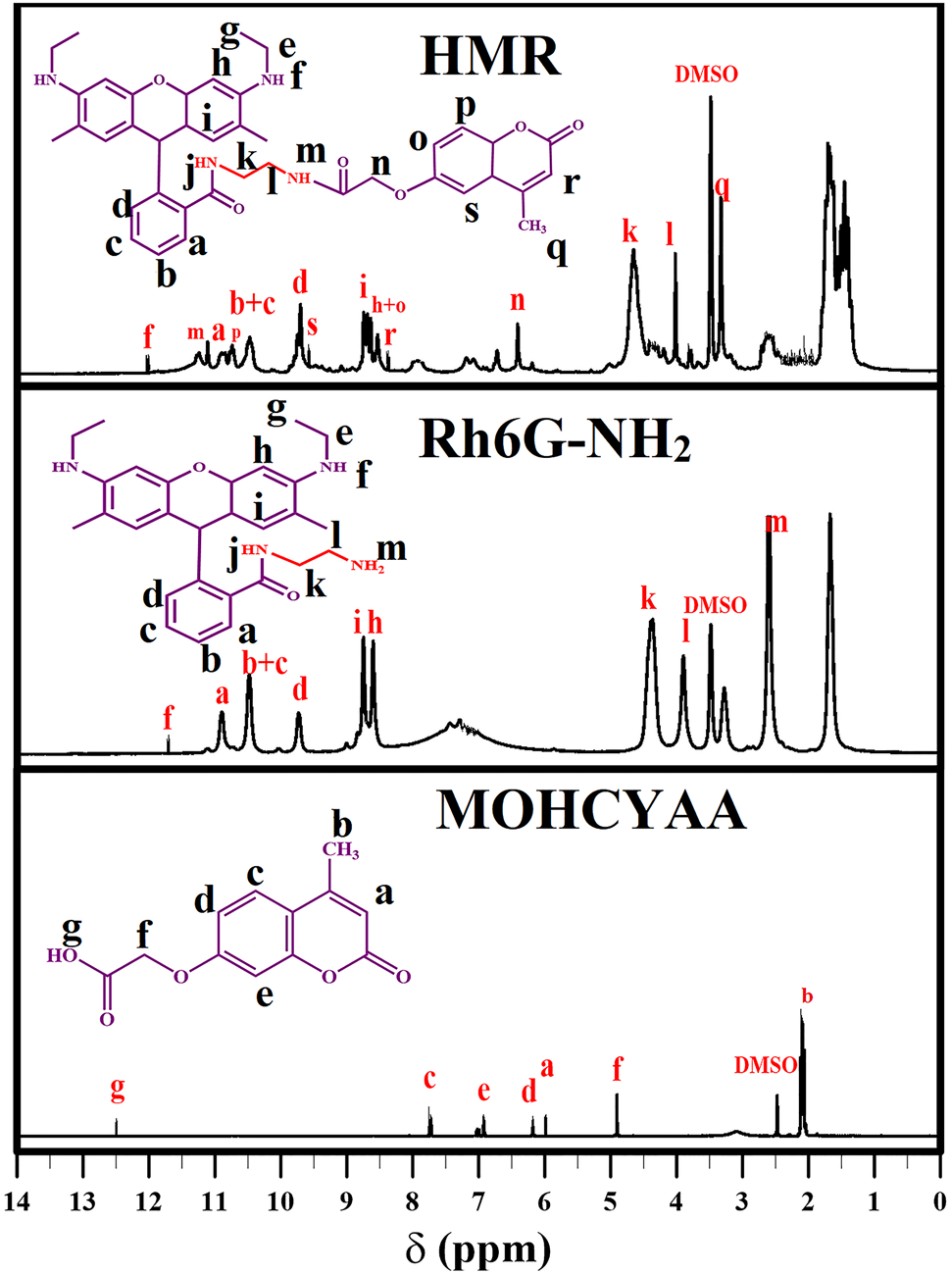
^1^H NMR spectra of MOHCYAA, Rh6G-NH_2_, and HMR.

^1^H NMR of Rh6G-NH_2_ (500 MHz, DMSO-*d*_6_, δ/ppm, Scheme S1, Fig. 2): 7.8 (m, 2H, a), 7.4 (m, 8H, b, c), 7.1 (s, 2H, d), 6.4 (m, 4H, i), 6.3 (m, 4H, h), 8.4 (m, 4H, f), 3.3 (m, 4H, k), 2.8 (m, 4H, l)^28^.

### Synthesis of 2-(4-methyl-2-oxo-2H-chromen-7-yloxy) acetic acid (MOHCYAA)

To prepare MOHCYAA pigment (Scheme 1), l g (5.67 mmol) 4-methyl-7-hydroxy coumarin, 4.25 g (30.75 mmol) anhydrous potassium carbonate, and 1.2 mL (10.8 mmol) ethyl bromoacetate in THF (23 mL) were refluxed for 16 h at 60 °C^29^. In the following, ethyl 2-(4-methyl-2-oxo-2 h-chromen-7-yloxy) acetate (0.5 g, 1 mmol) was dissolved in ethanol (13 mL). Then, 7 mL 5% NaOH solution was added to the reaction medium and the solvent was removed from the reaction medium. After dissolving the precipitate in water, 6 N HCl was added to the solution and allowed to form a white precipitate. Then, for purification, the precipitate was filtered using filter paper and separated from the solvent, and white-cream solid crystals were obtained by recrystallization from ethanol. Eventually, 2-(4-methyl-2-oxo-2H-chromen-7-yloxy) acetic acid was dried in a vacuum oven at room temperature for 24 h. Reaction yield was calculated ~ 60% gravimetrically.

FT-IR of MOHCYAA (KBr, cm^−1^, Fig. 1): (3400–3500 str. OH), (3068 CH str. C=CH), (2987, 2916 assym. str. CH_3_, CH_2_), (1755 C=O), (1708 C=O), (1610, 1566, 1510 str. C=C), (1427, 1390 CH_3_, CH_2_ bend), (1253 C–O), (1147 C–O)^29^.

^1^H NMR of MOHCYAA (500 MHz, DMSO-*d*_6_, δ/ppm, Scheme S2, Fig. 2): 2.3 (s, 3H, b), 4.6 (s, 2H, f), 6.0 (s, 1H, a), 6.2 (s, 1H, d), 6.9 (d, 1H, e), 7.6 (d, 1H, c)^29^.

### Synthesis of hybrid 2-(4-methyl-2-oxo-2H-chromen-7-yloxy) acetic acid/rhodamine 6G (HMR)

MOHCYAA (0.23 g, 0.1 mmol), DCC (0.028 g, 0.136 mmol), and DMAP (0.025 g, 0.1 mmol) were dissolved in DMF (20 mL). Then, Rh6G-NH_2_ (0.03 g, 0.07 mmol) in DMF (5 mL) was added dropwise to the reaction medium, and the mixture was stirred for 96 h at 25 °C under nitrogen (Scheme 1). After reaction completion, the product was washed with diethyl ether (40 mL) and dried at 60 °C for 48 h in a vacuum oven. Reaction yield was calculated ~ 80% gravimetrically^27^.

FT-IR of HMR (KBr, cm^−1^, Fig. 1): (2987, 2916 assym. str. CH_3_, CH_2_), (1755 C=O), (1708 C=O), (1610, 1566, 1510 str. C = C), 3400 (υNH), 1678 (C=O); 1634, 1528 and 1484 (Ar=CH)^15,27^.

^1^H NMR of HMR (500 MHz, DMSO-*d*_6_, δ/ppm, Scheme S3, Fig. 2): 7.8 (m, 2H, a), 7.4 (m, 8H, b, c), 7.1 (s, 2H, d), 6.3 (m, 4H, i), 6.2 (m, 4H, h), 8.6 (m, 4H, f), 3.4 (m, 4H, k), 2.8 (m, 4H, l), 4.7 (s, 2H, n), 6.2 (s, 1H, o), 7.6 (d, 1H, p), 2.3 (s, 3H, q), 6.0 (s, 1H, r), 6.8 (d, 1H, s)^15,27^.

## Results and discussion

### Synthesis of fluorescence hybrid dye

Hybrid dye was designed with rhodamine 6G covered with coumarin derivative. FT-IR, ^1^H NMR, ^13^C NMR, XRD, UV–vis-NIR, FS, FM, TGA, DLS, and FE-SEM analyses were used to evaluate and confirm in each step. The FT-IR and ^1^H NMR spectra of different pigments including COUM, MOHCYAA, Rh6G, Rh6G-NH_2_, and HMR pigments are depicted in Figs. 1 and 2, respectively and the most important peaks were mentioned in “Experimental methods” section. Also, ^13^C NMR spectra are shown in Fig. S1. To proof Rh6G modification process, different pigments were analyzed by TGA. Rh6G-NH_2_ and HMR thermograms are shown in Fig. 1. The degradation temperature (T_d,max_) and weight loss of pigments were obtained 345 °C and 69.0% for Rh6G-NH_2_, 352 °C and 64.5% for HMR. X-ray diffraction patterns were collected to confirm the crystal structure of samples and investigate how crystallinity is affected by different reactions^30^. XRD patterns of all samples are shown in Fig. 1. Coumarin possesses peaks at 2θ = 15.8°, 19.9°, 22.4°, 25.3°, 27.6°, 29.8°, 36°, and 37.8. In addition, the exhibited peaks of Rh6G at 2θ = 13.5°, 15.4°, 18.9°, 17.7°, 19.62°, 24.9°, and 26.9° are in agreement with the literature^31^. All pigments contain amorphous and crystalline phases, while the amount of these phases varies for different compounds. Crystallinity index of pigments was obtained according to Eq. (1) ^15^.

1 $${\text{Crystallinity (\% ) = }}\frac{{\text{Total area of crystalline peaks}}}{{\text{Total area of all peaks}}} \, \times {100}$$

The crystallinity of pigments was obtained 50.9, 79.2, 55.6, 48.5, and 64.8% for Rh6G, COUM, MOHCYAA, Rh6G-NH_2_, and HMR, respectively.

**Figure 3:**
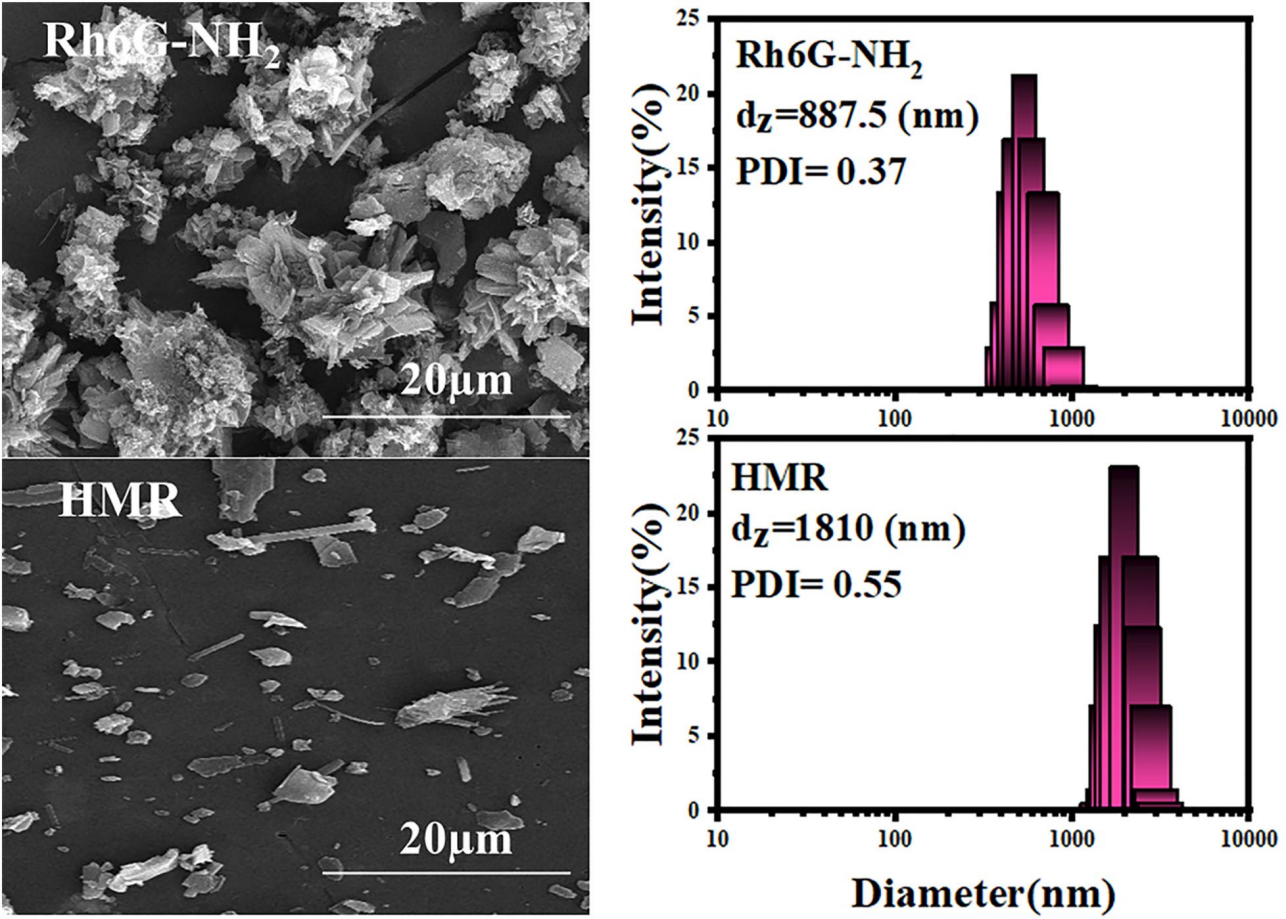
FE-SEM images and DLS results of Rh6G-NH_2_ and HMR.

Figure 3 shows FE-SEM images and DLS results of Rh6G-NH_2_ and HMR. The pigment structure of Rh6G-NH_2_ is small aggregates resembling flake particles with small sections. After reaction of Rh6G-NH_2_ with MOHCYAA, almost spherical particles were observed. DLS was performed to investigate the changes in size of samples after each step. To this end, a 1 mg/mL solution of Rh6G-NH_2_ and HMR were analyzed at 25 °C. Z-average particle size of Rh6G-NH_2_ and HMR were reported 887.5 and 1810 nm, respectively. The PDI values for Rh6G-NH_2_ and HMR were 0.37 and 0.55, respectively.

### Photophysical properties

**Scheme 2:**
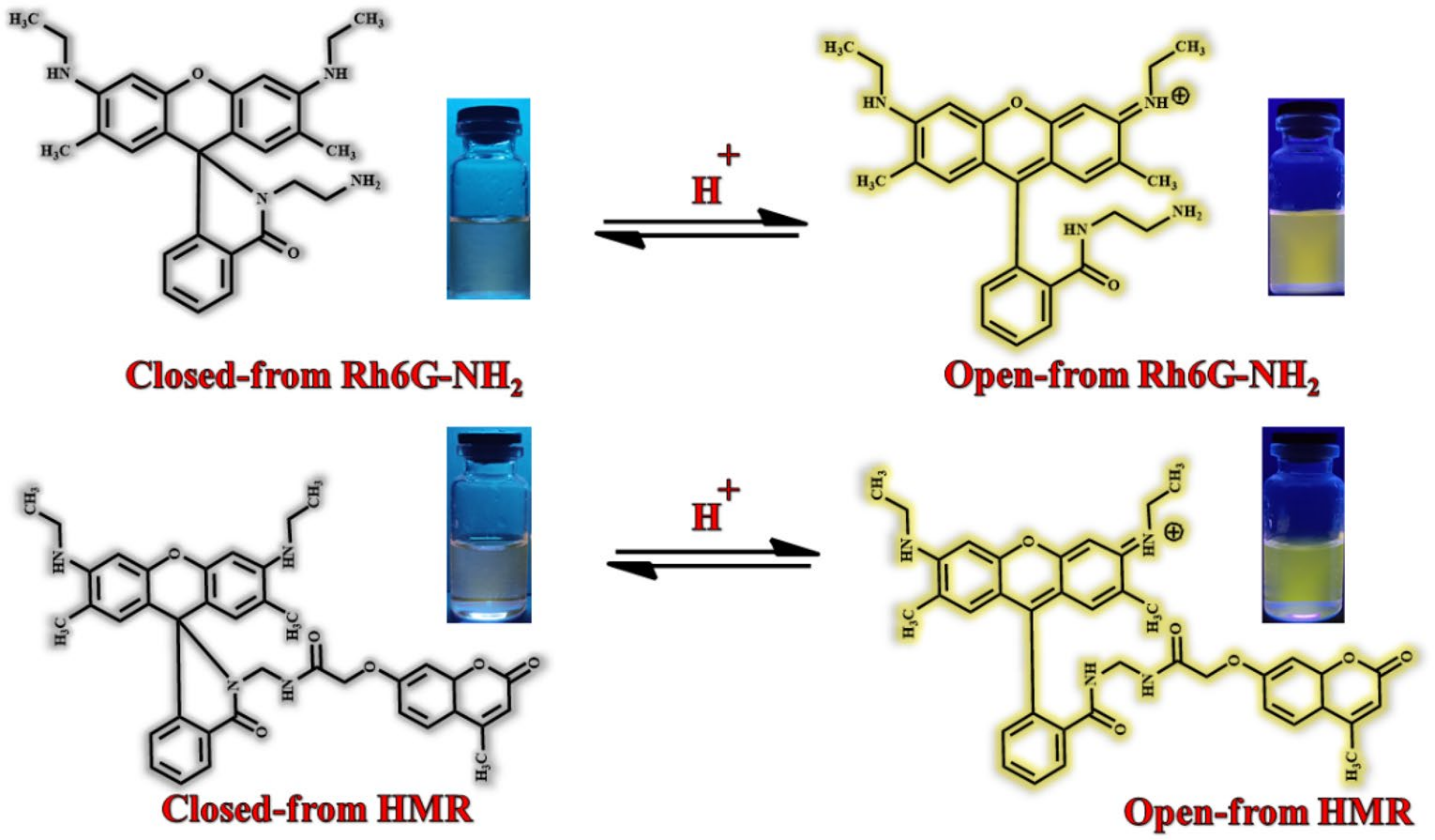
Mechanism of emission rhodamine6G derivatives.

A bichromophoric light-harvesting system was designed, including a coumarin donor and a rhodamine 6G receptor. Due to the pH-sensitive nature of rhodamine 6G, we expect new fluorescence signals. Behavior of Rh6G-NH_2_ and HMR pigments depends on pH. In alkaline solution, rhodamine 6G derivatives are colorless in the form of spirolactam closed form. In acidic environment, the rhodamine 6G spirolactam ring is opened and the energy of coumarin in HMR pigment is transferred to rhodamine 6G and emits a yellow-green fluorescence signal (Scheme 2).

Photophysical properties of pigments in H_2_O, DMF, and ethanol were investigated and results are summarized in Table 1. MOHCYAA, Rh6G-NH_2_, and HMR pigments were used as model pigments to evaluate the photophysical properties. Using Eq. (2) ^32^, the fluorescence quantum efficiency of pigments was calculated.

2 $$\Phi_{s} = \Phi_{r} \frac{{m_{s} }}{{m_{r} }}\left( {\frac{{\eta_{s} }}{{\eta_{r} }}} \right)^{2}$$

**Table 1:** Photophysical properties of pigments.

Sample	*λ*_*ex*_^*a*^	*λ*_*em*_^b^	*λ*_*max*_^c^	Solvent	*Φ*_*s*_^d^	Stoke shift (nm)
MOHCYAA	324	380	324	H_2_O	0.34	56
	324	380	324	DMF	0.30	56
	324	380	324	Ethanol	0.01	56
	324	380	324	pH1	0.15	56
	324	380	324	pH7	0.005	56
	324	380	324	pH10	0.003	56
	318	388	244,318	E-pH1^e^	0.22	70
	319	389	319	E-pH7	0.12	70
	315	388	315,276	E-pH10	0.14	73
Rh6G-NH_2_	525	540	524	H_2_O	0.41	16
	525	555	490,525	DMF	0.11	15
	525	540	524	Ethanol	0.66	16
	525	550	525	pH1	0.29	25
	525	540	525	pH7	0.12	16
	525	540	525	pH10	0.07	16
	525	579	530	E-pH1	0.52	49
	525	579	527	E-pH7	0.41	52
	525	579	516	E-pH10	0.34	63
HMR	305	390,558	305,527	H_2_O	0.37	31
	305	390,669	305,527	DMF	0.19	142
	305	390,548	305,527	Ethanol	0.72	21
	305	390,552	305,527	pH1	0.18	25
	305	390,552	305,527	pH7	0.10	25
	305	390,552	305,527	pH10	0.08	25
	232	393,574	232,530	E-pH1	0.59	44
	299	393,574	299,525	E-pH7	0.48	49
	316	393,574	281,316	E-pH10	0.40	49

^a^Excitation wavelength, ^b^maximum emission wavelength, ^c^maximum absorption wavelength, ^d^fluorescence quantum yield, ^e^pH in ethanol.

In this equation, *Φ*_*r*_ is the quantum yield of the standard pigment, *m*_*s*_ is the slope of the linear fit for the integrated fluorescence intensity of the fluorescent pigment as a function of absorbance, and *η*_*s*_ and *η*_*r*_ are the refractive index of the fluorescent pigment and the standard solutions, respectively. Figures S2, S3, 4, 5, 6, and 7 show the absorption and emission spectra for COUM, Rh6G, MOHCYAA, Rh6G-NH_2_, and HMR in different solvents. The strongest absorption band of MOHCYAA was observed at 324 nm in H_2_O, DMF, ethanol, pH = 1, pH = 7, pH = 10, and in ethanol of pH = 1, pH = 7, and pH = 10. MOHCYAA showed blue emission with emission spectra at 380 nm with fluorescence quantum efficiencies of *Φ*_*s*_ = 0.34, 0.30, 0.01, 0.15, 0.005, and 0.003 in H_2_O, DMF, ethanol, pH = 1, pH = 7, and pH = 10, respectively. The strongest absorption band of Rh6G-NH_2_ was observed at 524 nm in H_2_O, DMF, ethanol, pH = 1, pH = 7, and pH = 10. Rh6G-NH_2_ showed yellow emission with emission spectra at 555 nm with fluorescence quantum efficiencies of *Φ*_*s*_ = 0.41, 0.11, 0.66, 0.29, 0.12, and 0.07 in H_2_O, DMF, ethanol, pH = 1, pH = 7, and pH = 10, respectively. After modification of Rh6G-NH_2_ with MOHCYAA, maximum absorption peaks (Fig. 6) were shifted to 305 and 527 nm in H_2_O, DMF, ethanol, pH = 1, pH = 7, and pH = 10. Conforming to Eq. (2), fluorescence quantum yield (*Φ*_*s*_) of HMR was calculated 0.37, 0.19, 0.72, 0.18, 0.1, and 0.08 in H_2_O, DMF, and ethanol, pH = 1, pH = 7, and pH = 10, respectively. Moreover, different spectra differ in wavelength of peak and its intensity. General trends of spectra in normalized form considering intensity and peak wavelength are similar. This shows that the molecular physics governing the fluorescence process is not significantly affected by solvent. Observed deflections in different spectra can be related to slight differences in the solubility of Rh6G and coumarin molecules in individual solvents.

**Figure 4:**
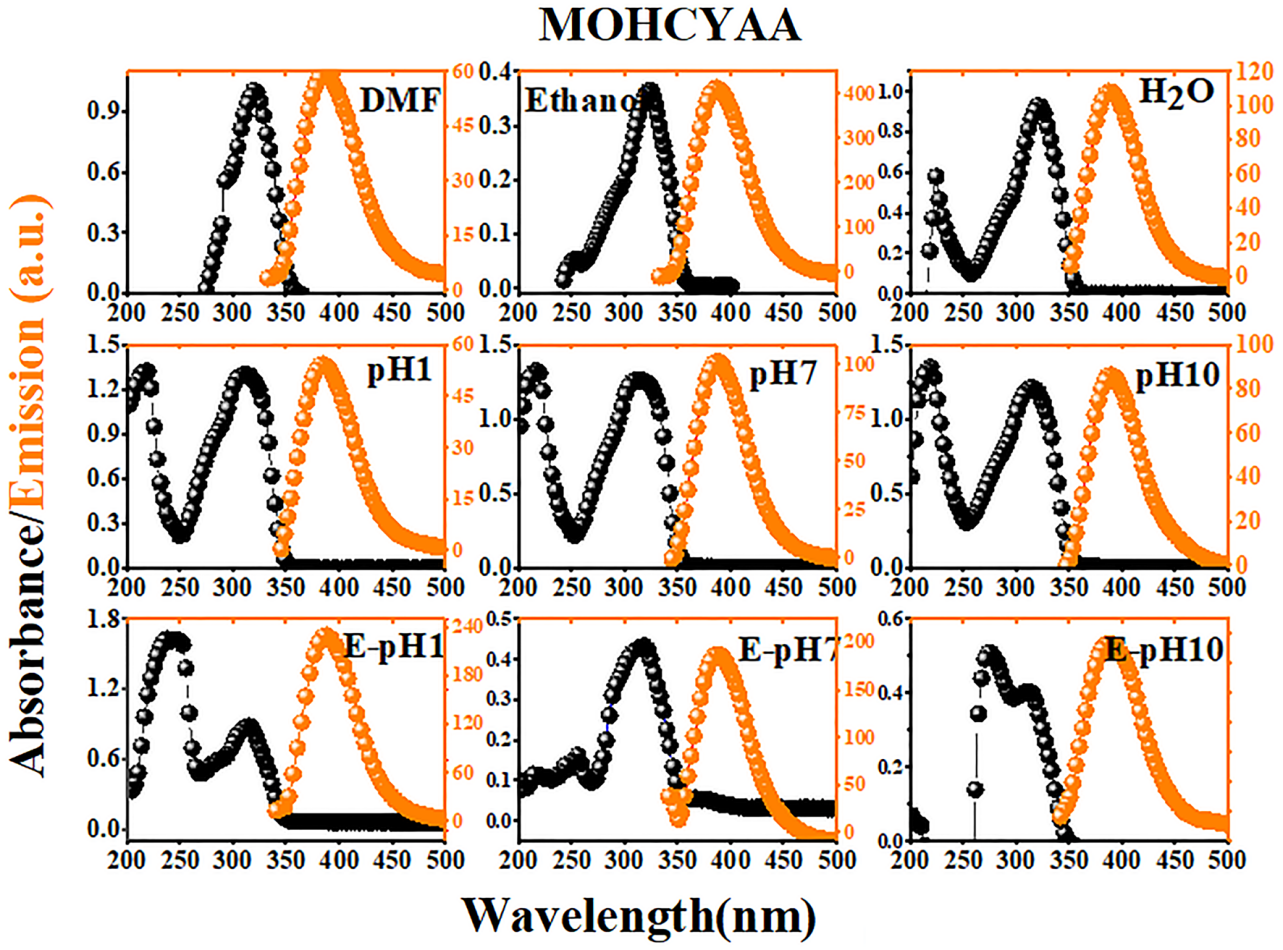
Normalized absorption and fluorescence spectra of MOHCYAA in different solvents.

**Figure 5:**
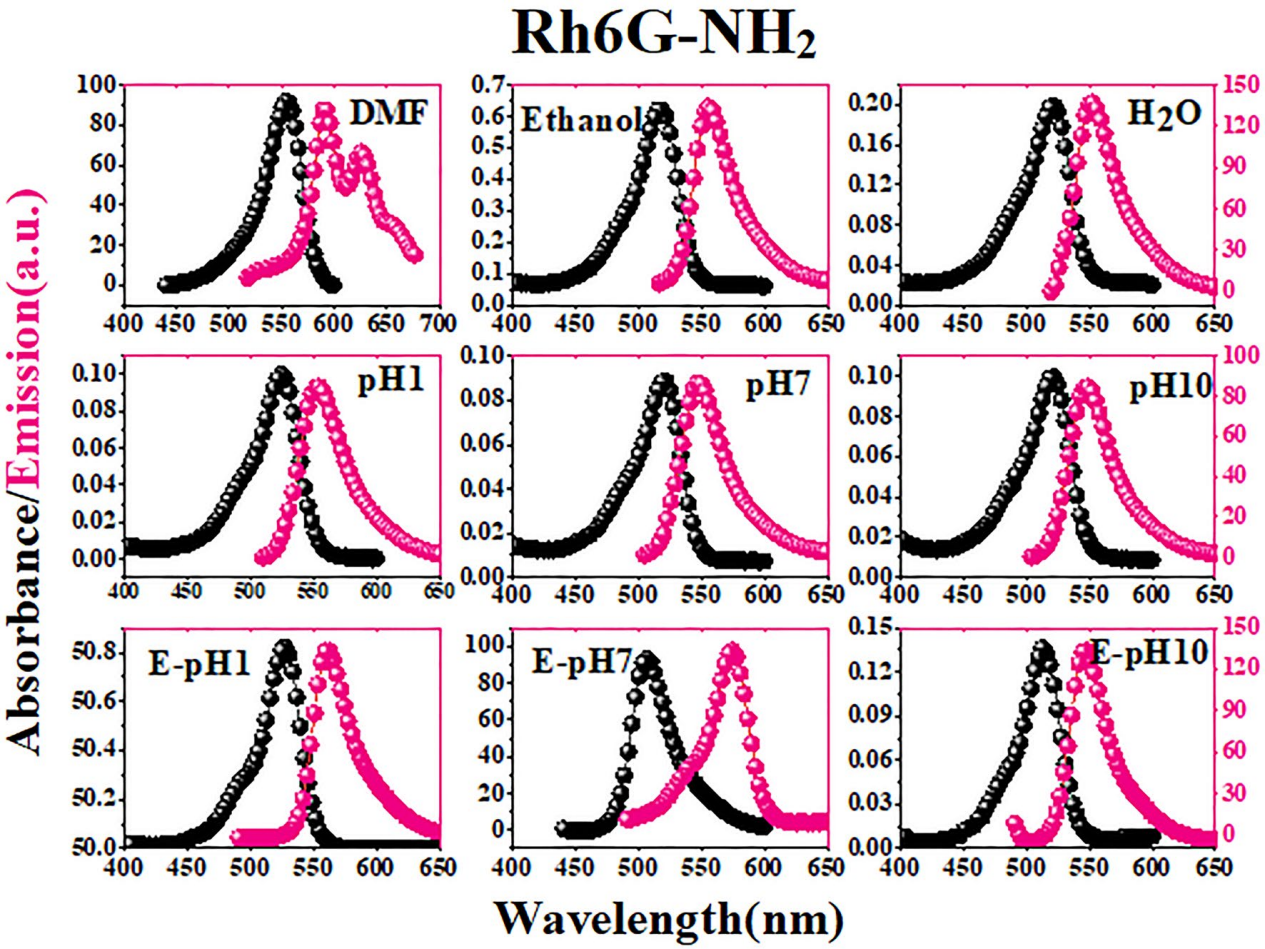
Normalized absorption and fluorescence spectra of Rh6G-NH_2_ in different solvents.

**Figure 6:**
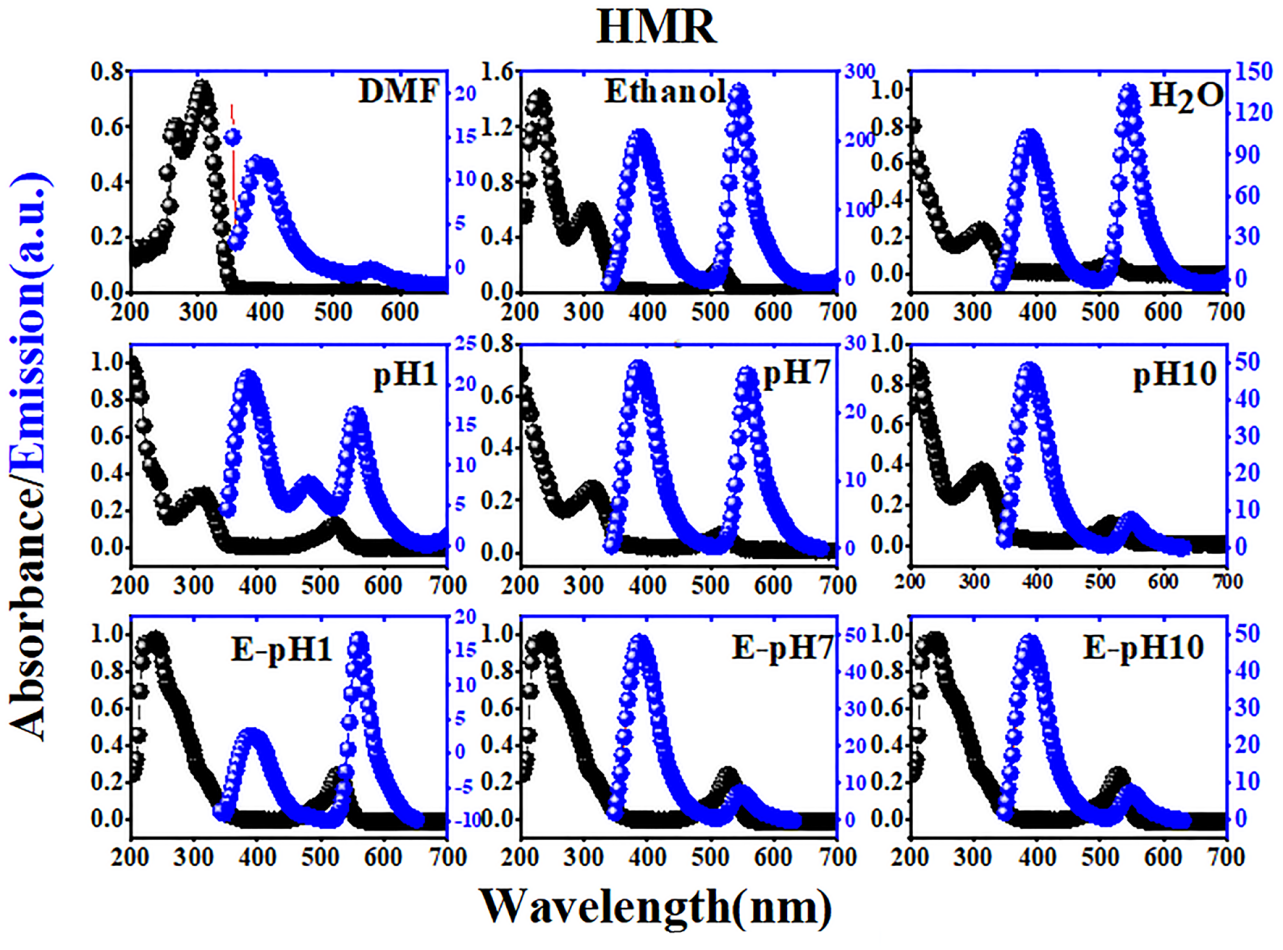
Normalized absorption and fluorescence spectra of HMR in different solvents.

**Figure 7:**
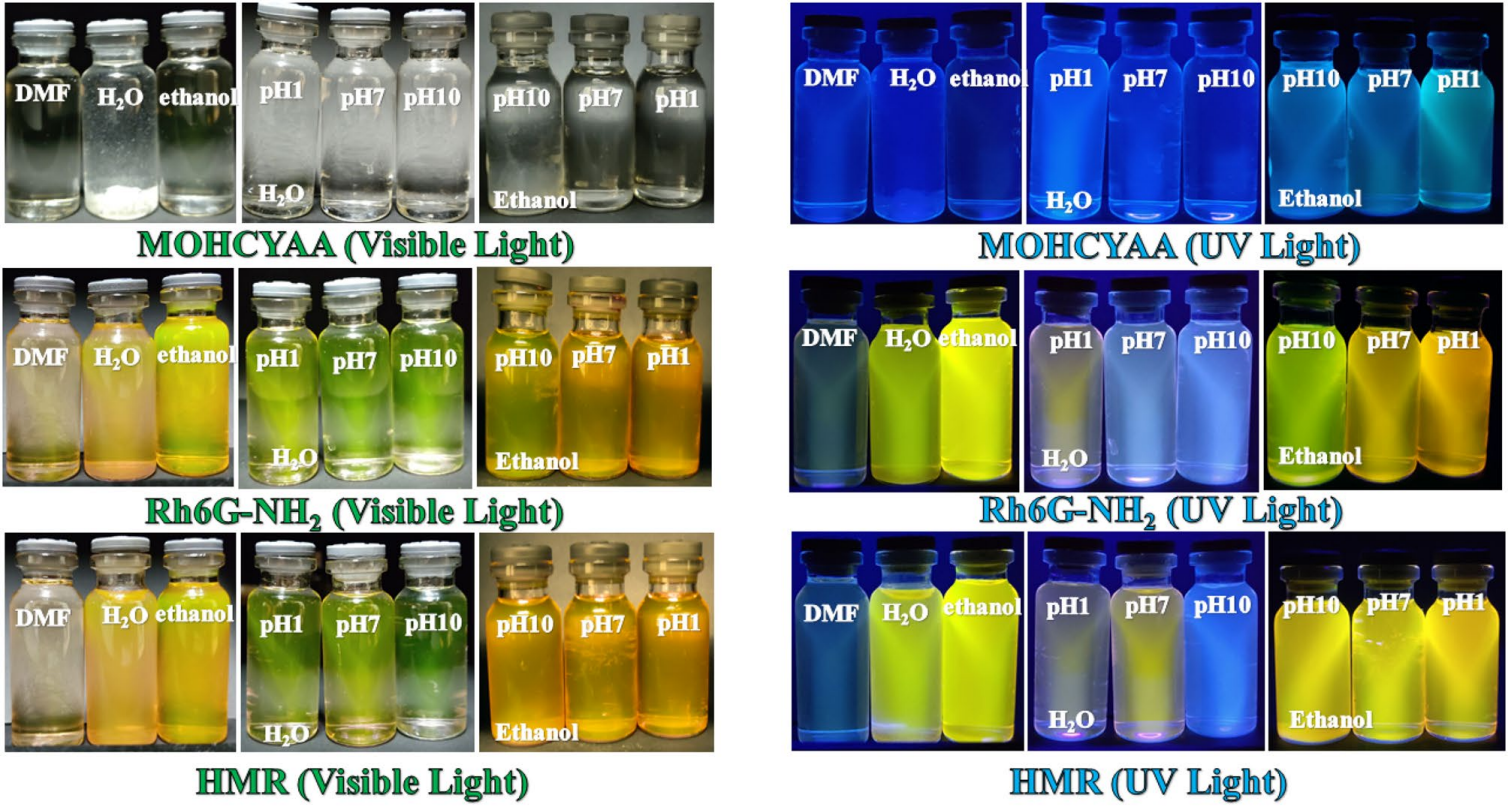
Solution of MOHCYAA, Rh6G-NH_2,_ and HMR in various solvents.

**Figure 8:**
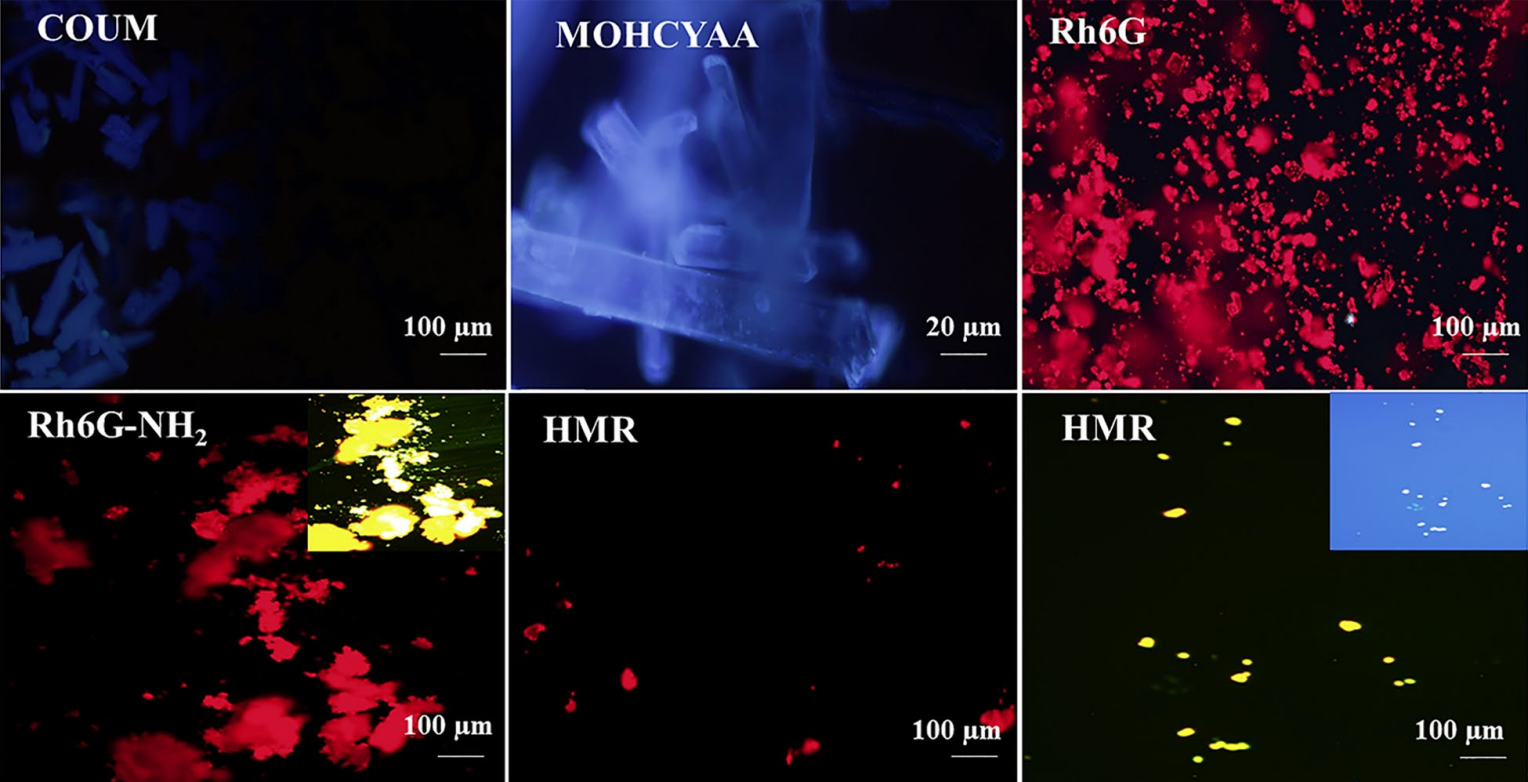
Fluorescence microscopy images of COUM, MOHCYAA, Rh6 G, Rh6G-NH_2_, and HMR.

Figure 8 shows the fluorescence images of COUM, MOHCYAA, Rh6G, Rh6G-NH_2_, and HMR. Owing to the red and yellow emission of rhodamine6G derivatives and the blue emission of coumarin derivatives, synthesized hybrid samples were evaluated by using two and three filters for Rh6G-NH_2_ and HMR, respectively. Stability of the fluorescence emission of Rh6G-NH_2_ in red and green and HMR in red, green, and blue fluorescence filters is quite evident.

### UV–visible–NIR reflectance properties

**Figure 9:**
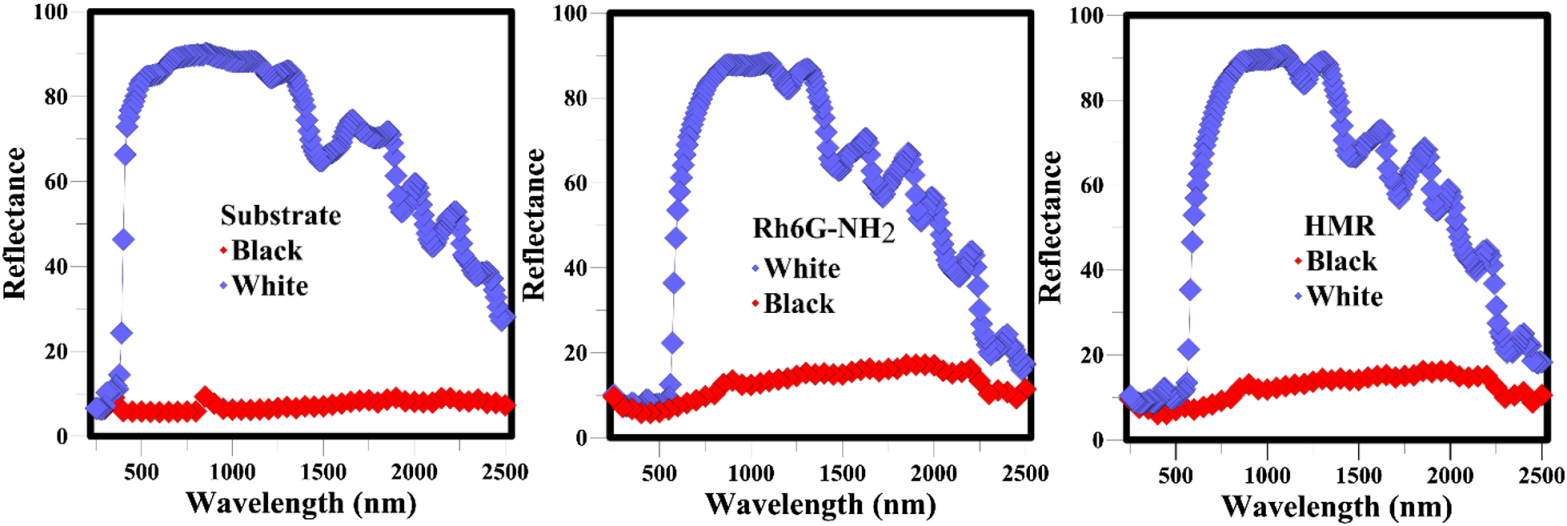
UV–Vis-NIR spectra of Rh6G-NH_2_ and HMR.

Figure 9 shows the UV–vis-NIR spectra of Rh6G-NH_2_ and HMR in the wavelengths of 250–2500 nm. Reflection of pigments in the visible region is slightly different due to differences in their color^33^. The colors absorb the most ultraviolet light, which is in line with the organic nature of these colors and causes a similar reflection in the ultraviolet region. In addition, colors created different reflections on the white and black backgrounds. The reflection rate on the black and white substrates was < 20% and > 70%, respectively. Therefore, Rh6G-NH_2_ and HMR are classified as transparent pigments in NIR area^34^. Furthermore, integrals of Rh6G-NH_2_ and HMR pigments in different regions of UV–Vis-NIR curves were investigated and the data are summarized in Table 2. Rh6G-NH_2_ and HMR had reflection of 95.4 and 95.4% in range of 700–1000 nm, respectively, and showed very good transparency.

**Table 2:** Investigation of percentage of reflection of Rh6G-NH_2_ and HMR in black and white substrates.

Sample	700–1000 (nm)	1000–1500 (nm)	1500–2500 (nm)
White substrate	26,819	41,188	55,577
Rh6G-NH_2_	25,605	40,664	47,014
HMR	25,605	40,664	47,014

Sample	Reflection (%)	Reflection (%)	Reflection (%)
Rh6G-NH_2_	95.4%	98.7%	84.6%
HMR	95.4%	98.7%	84.6%

Sample	700–1000 (nm)	1000–1500 (nm)	1500–2500 (nm)
Black substrate	1981	3297	8223
Rh6G-NH_2_	3460	7099	14,800
HMR	3314	6730	14,065

Sample	Reflection (%)	Reflection (%)	Reflection (%)
Rh6G-NH_2_	174.6%	215.3%	179.9%
HMR	167.2%	204.1%	171.0%

## Conclusions

Rh6G was modified by EDA to obtain Rh6G-NH_2_. Rh6G-NH_2_ as an initial core was used to bond coumarin derivatives. ^1^H NMR, FT-IR, XRD, TGA, FE-SEM, visible ultraviolet, Fluorescence spectrophotometer, DLS, and UV–Vis-NIR reflectance were used to confirm the success of various processes. Photophysical properties of MOHCYAA, Rh6G-NH_2_, and HMR were investigated. Type of solvent had a strong effect on quantum yield. Rh6G-NH_2_ (*ϕ*_*s*_ = 0.66) and HMR (*ϕ*_*s*_ = 0.72) displayed the maximum quantum yield in ethanol due to good interaction with ethanol and the formation of ring-opened amide form of rhodamine group. UV–Vis-NIR reflectance spectra showed that Rh6G-NH_2_ and HMR had a reflectance of 95.4% and 95.4% in 700–1000 range, respectively. As a result, Rh6G-NH_2_ and HMR showed good transparency.

## Supplementary Information

Supplementary Information.

## Data Availability

The datasets generated and/or analyzed during the current study are not publicly available at this time as the data form part of an ongoing study. However, the datasets are available from the corresponding author (Mehdi Salami-Kalajahi, m.salami@sut.ac.ir) on reasonable request.
