# Supplementary material for: Reflectance and photophysical properties of rhodamine 6G/2-(4-methyl-2-oxo-2H-chromen-7-yloxy) acetic acid as cold hybrid colorant
Source: Sci Rep. 2022 Apr 12;12:6145. doi: 10.1038/s41598-022-10001-9 (PMC9005515; doi:10.1038/s41598-022-10001-9)
Supplement: Supplementary file 1 — Supplementary Information. [file 41598_2022_10001_MOESM1_ESM.docx]

**Supporting Information for**

**Reflectance and photophysical properties of rhodamine 6G/2-(4-methyl-2-oxo-2H-chromen-7-yloxy) acetic acid as cold hybrid colorant**

Behnam Gheitarani^1,2^, Marzieh Golshan^1,2^, Mahdi Salami Hosseini^1,2,*^, Mehdi Salami-Kalajahi^1,2,^*

^1^ Faculty of Polymer Engineering, Sahand University of Technology, P.O. Box 51335-1996, Tabriz, Iran

^2^ Institute of Polymeric Materials, Sahand University of Technology, P.O. Box 51335-1996, Tabriz, Iran

* Correspondence concerning this article should be addressed to

Mahdi Salami Hosseini: Email : salami@sut.ac.ir, Tel. /Fax : +98 41 33459082

Mehdi Salami-Kalajahi : Email : m.salami@sut.ac.ir, Tel. /Fax : +98 41 33459097

S1. Experimental Methods

S1.1. Materials

Rhodamine 6G (Rh6G, Aldrich, 95%), sodium hydroxide (NaOH, Dr. Mojallali, 95%), methanol (Merck, 99.9%), tetrahydrofuran (THF, Merck, 99%), ethylenediamine (EDA, Aldrich, 99%), nitrocellulose resin, *N*,*N*-dimethylformamide (DMF, DaeJung, 99.5%), hydrochloric acid (HCl, Dr. Mojallali, 37%), 4-methylumbelliferone (COUM, Aldrich, 99.5%), potassium carbonate (K_2_CO_3,_ Dr. Mojallali, 99.5%), 4-(dimethylamino)pyridine (DMAP, Aldrich, 99%), ethyl bromoacetate (Aldrich, 97%), N,N*′-*dicyclohexylcarbodiimide (DCC, Merck, 99%), and dye thinner (Dr. Mojallali, 20000) were used as received.

S1.2. Dye preparation

Nitrocellulose resin (64 *wt*. %), pigments (16 *wt*. %), and solvent (20 *wt*. %, dye thinner) were mixed and ball-milled for 5 h. Then, resultant paints were applied to Leneta checkerboard charts using a film applicator with a thickness of 120 µm. This helped to prevent reflection of the substrate and only reflection of paint was detected. Evaluation of pigments reflectance on a highly reflective substrate (white substrate) and highly absorbent substrate (black substrate) can qualitatively show the amount of reflection, transfer, and absorption of pigment.

S1.3. Instrumentation

Fourier transform infrared (FT-IR) spectroscopy was recorded on a Bruker Tensor 27 FT-IR spectrophotometer in the wavenumber range of 500 to 4000 cm^−1^ with a resolution of 4 cm^−1^. An average of 24 scans were performed for each sample prepared on a KBr pellet in vacuum desiccators.

The Varian Unity Inova model (500 MHz) proton and carbon nuclear magnetic resonance (^1^H and ^13^C NMR) were used to verify accuracy of synthesis in each step using deuterated DMSO (DMSO-*d*_6_) as solvent.

Fluorescence microscopy (FM, BEL, Model: FLUO-3) with a camera (BLACKL. 5000 MET) was used to determine the fluorescence properties of PTCDI derivatives.

Field emission scanning electron microscopy (FE-SEM) was performed using a TESCAN MIRA3 (cold field emission scanning electron microscope) on a work voltage of 30 kV to observe the morphology of rhodamine and coumarin derivatives. The powder samples were coated with gold before the characterization.

Size and size distribution of samples were measured by a DLS instrument (Malvern Nano Zetasizer ZS 90, UK) with a scattering angle of 176.1°.

Ultraviolet visible (UV–visible) absorption spectra from liquid samples were recorded by a Hanon instrument.

Fluorescence spectrophotometer (FS, PerkinElmer LS-45) was used to determine the emission rate of pigments.

X-ray diffraction (XRD) was used to investigate the structural properties of produced films. XRD spectra were collected on an X-ray diffraction instrument (Siemens D5000) with a Cu target (λ = 0.1540 nm) at room temperature. The system consists of a rotating anode generator which operated at 35 kV and 20 mA. The samples were scanned from 2θ = 10 to 40° at the step scan mode; the diffraction pattern was recorded using a scintillation counter detector.

UV–Vis–NIR reflectance spectra were obtained over the wavelength range of 250–2500 nm using a PerkinElmer lambda 1050.

Thermal gravimetric analyses (TGA) were carried out by means of a PL thermo-gravimetric analyzer (Polymer Laboratories, TGA 1000, UK). The pigments (about 10 mg) were heated from ambient temperature to 700 °C at a heating rate of 10 °C/min and nitrogen as the purging gas was used at a flow rate of 50 mL/min.

S2. Results





**Scheme S1: Rh6G-NH_2_ structure**





**Scheme S2: MOHCYAA structure**





**Scheme S3: HMR structure**


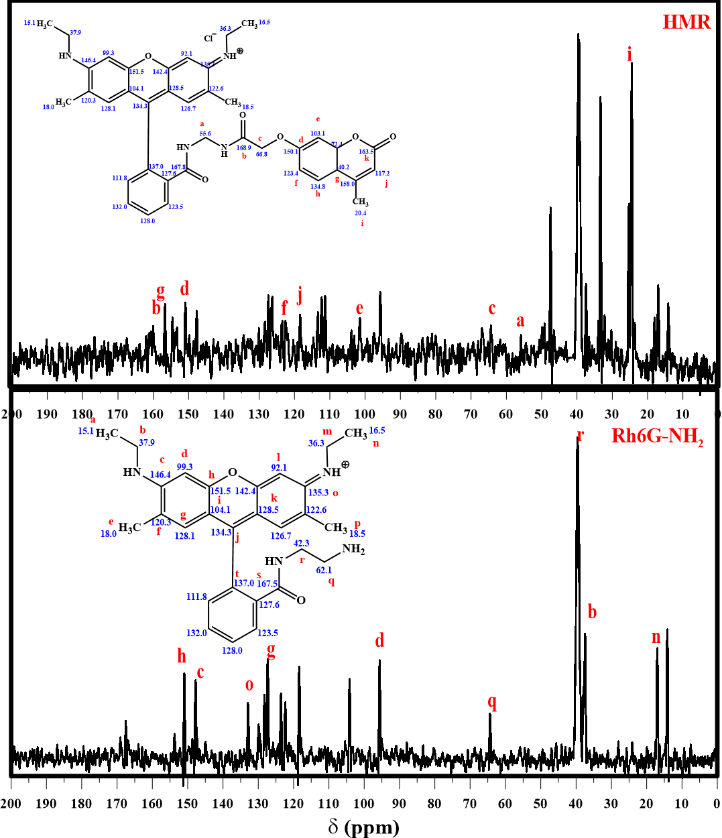


**Figure S1. ^13^C NMR spectra of Rh6G-NH_2_ and HMR**

**
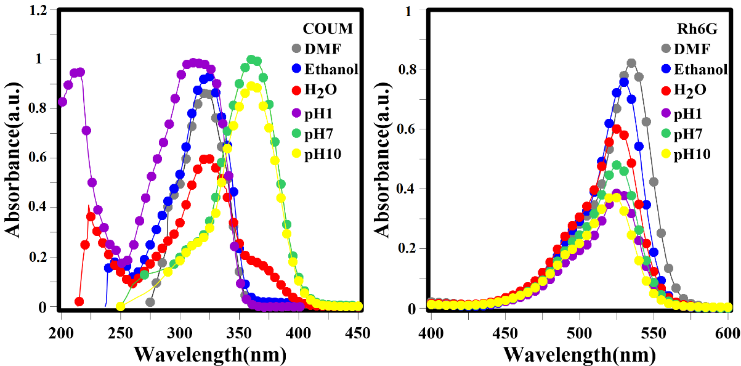
**

**Figure S2: Absorption spectra of COUM and Rh6G**

**
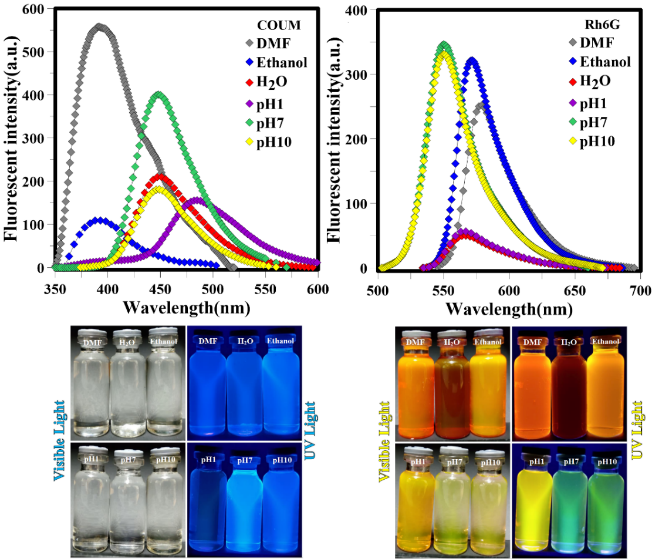
**

**Figure S3: Fluorescence spectra of COUM and Rh6G**

**Table S1: Photophysical properties of pigments**

| **Sample** | ***λ_ex_^a^*** | ***λ_em_*^b^** | ***λ_max_*^c^** | **Solvent** | ***Φ_s_*^d^** |
| --- | --- | --- | --- | --- | --- |
| **COUM** | 327  325  327  315  365  365 | 450  390  390  480  450  450 | 327  325  327  315  365  365 | H_2_O  DMF  Ethanol  pH1  pH7  pH10 | 0.35  0.13  0.21  0.09  0.01  0.01 |
| **Rh6G** | 524  527  525  524  524  524 | 565  575  565  565  540  540 | 524  527  525  524  524  524 | H_2_O  DMF  Ethanol  pH1  pH7  pH10 | 0.10  0.23  0.95  0.54  0.22  0.13 |
